# Ultrasonic-Assisted Extraction of Polysaccharides from *Schizochytrium limacinum* Meal Using Eutectic Solvents: Structural Characterization and Antioxidant Activity

**DOI:** 10.3390/foods14111901

**Published:** 2025-05-27

**Authors:** Xinyu Li, Jiaxian Wang, Guangrong Huang, Zhenbao Jia, Manjun Xu, Wenwei Chen

**Affiliations:** College of Life Sciences, China Jiliang University, Hangzhou 310018, China; 15990179129@163.com (X.L.); 18868432919@163.com (J.W.); 07b0904052@cjlu.edu.cn (G.H.); zhenbaojia@cjlu.edu.cn (Z.J.); 18258352895@163.com (M.X.)

**Keywords:** ultrasonic-assisted eutectic solvent extraction, polysaccharides, antioxidant activity, *Schizochytrium limacinum* meal, structural characterization

## Abstract

To address the underutilization of *Schizochytrium limacinum* meal, polysaccharides from *Schizochytrium limacinum* meal (SLMPs) were prepared via ultrasonic-assisted eutectic-solvent-based extraction. Although polysaccharides exhibit promising application potential, the structural ambiguity of SLMPs necessitates systematic investigation to elucidate their structure–activity relationships, thereby providing a scientific foundation for their subsequent development and utilization. Using response-surface methodology (RSM), the optimized extraction conditions were determined as follows: ultrasonic temperature of 52 °C, ultrasonic duration of 31 min, ultrasonic power of 57 W, water content of 29%, and a material-to-liquid ratio of 1:36 g/mL. Under these conditions, the maximum polysaccharide yield reached 9.25%, demonstrating a significant advantage over the conventional water extraction method (4.18% yield). Following purification, the antioxidant activity and structural characteristics of SLMPs were analyzed. The molecular weight, monosaccharide composition, reducing groups, and higher-order conformation were systematically correlated with antioxidant activity. Fourier-transform infrared spectroscopy (FTIR), monosaccharide composition analysis, and ^1^H nuclear magnetic resonance (NMR) spectroscopy revealed characteristic polysaccharide functional groups (C–O, O–H, and C=O). Monosaccharides such as glucose (Glc), galactose (Gal), and arabinose (Ara) were found to enhance antioxidant activity. High-performance gel permeation chromatography (HPGPC) indicated a molecular weight of 20.7 kDa for SLMPs, with low-molecular-weight fractions exhibiting superior antioxidant activity. Scanning electron microscopy (SEM) further demonstrated that ultrasonically extracted polysaccharides possess porous structures capable of chelating redox-active functional groups. These findings suggest that ultrasonic-assisted eutectic-solvent-based extraction is an efficient method for polysaccharide extraction while preserving antioxidant properties.

## 1. Introduction

*Schizochytrium limacinum* is a high-value, safe, and efficient single-celled marine microalga with advantages such as an extremely fast growth rate and ease of cultivation while accumulating large amounts of nutrients like lipids, proteins, and polysaccharides during growth [1,2,3]. Due to the rich docosahexaenoic acid (DHA) content of *S. limacinum*, it is widely utilized for the extraction of DHA algal oil [4]. However, after the extraction of algal oil, the remaining *S. limacinum* meal is underutilized; only a small portion is used as feed or fertilizer, and most of it is discarded. The outcomes are resource wastage and environmental pollution. With increased emphasis on sustainable development and environmental conservation, green extraction methods for natural bioactive substances have become a research focus. Therefore, recycling polysaccharides from *S. limacinum* meal can help in the implementation of the green development concept.

Polysaccharides possess various health-related functional activities, such as antioxidant [5], anti-inflammatory [6], antitumor [7], and immunomodulatory effects [8]. Traditional techniques such as hot water extraction, acid–base treatment [9,10], and alcohol extraction [11] have a common problem of low extraction efficiency. In recent years, the development of new extraction technologies has significantly improved extraction efficiency, mainly including innovative methods such as microwave-assisted extraction [12], ultrasound-assisted extraction [13], enzyme extraction [14], and the deep eutectic solvent (DES) method [15]. Among them, ultrasound-assisted extraction (UAE) has attracted much attention due to its simple operation and significant efficiency—the mechanical vibration generated by the cavitation effect can effectively break down the cell structure, while enhancing the solvent vortex diffusion effect, thereby promoting the efficient dissolution of polysaccharide components [16]. However, it should be noted that the physical effects generated during ultrasonic treatment may cause structural damage to polysaccharides. The use of DESs represents a breakthrough in green chemistry, characterized by nontoxicity, economy, environmental friendliness, biodegradability, nonflammability, and stability [17]. Establishing an ultrasonic-assisted DES-based method (DES-UAE) contributes to the effective utilization, quality control, and bioactivity exploration of polysaccharides from *S. limacinum* meal. This method reduces the use of harmful solvents, saving cost and time. It also significantly improves the extraction of active polysaccharides due to the cavitation effect, meeting industrial-scale production requirements.

The present study aimed to enhance the resource-utilization efficiency of *S. limacinum* meal. A DES-UAE method was established and optimized using the Box–Behnken experimental design (BBD) to improve extraction efficiency. The obtained crude polysaccharides were separated and purified to explore their structural characterization and understand their structure–activity relationships. To enable the polysaccharides derived from alga meal to be widely used in food and medicine, their antioxidant activity was assessed as well.

## 2. Materials and Methods

### 2.1. Materials

The Chinese company Wuxi Green Flag Biotechnology Company provided the *S. limacinum* meal. The meal was dried and ground to obtain algal powder.

The algal powder was packaged in filter paper and secured with a thin rope. Using petroleum ether as the solvent, the algae powder was subjected to reflux degreasing using the Soxhlet extraction method for 7 h. Then, the obtained powder was dried in a 45 °C, atmospheric pressure oven for 3 h.

### 2.2. Low Eutectic Solvents and Ultrasonic-Assisted Extraction Method

DESs were prepared according to a previous methodology [18]. Five different hydrogen-bond donors were selected and mixed thoroughly with the hydrogen-bond acceptor choline chloride in specific molar ratios at 80 °C to obtain homogeneous, stable, and transparent solutions (Table 1).

To select the best DESs, the algal powder was mixed with various DESs. The *S. limacinum* meal polysaccharides (SLMPs) were extracted using the UAE method (30% water content, 50 °C, 30 min, 70 W ultrasonic power). Polysaccharide content was determined by the phenol–sulfuric acid method after centrifugation [19]. The best DES was compared and selected.

The following formula was used to obtain the SLMP yield:

(1) $$Yield\left(\%\right)=\frac{the\;dry\;weight\;of\;SLMPs}{the\;weight\;of\;Schizochytrium\;Limacinum\;meal}\;\;\;$$

### 2.3. Single-Factor Experiment

Using the best DES selected, we optimized the DES-UAE method. The five most important influencing factors were investigated through one-way experiments: ultrasonic temperature (30–70 °C), ultrasonic time (10–50 min), ultrasonic power (40–80 W), DES water content (10–50%, *v*/*v*), and material/liquid ratio (1:20 to 1:40, g/mL).

### 2.4. Response-Surface Experimental Design

We selected five factors, namely ultrasonic temperature (A), ultrasonic time (B), ultrasonic power (C), water content (D), and material/liquid ratio (E), at three levels each, resulting in 46 experimental runs. The experiments were designed and analyzed using Design-Expert software (version 8.0) to obtain the optimized conditions for SLMP extraction.

### 2.5. Separation and Purification

The extracted polysaccharides were added to 4 volumes of Sevag reagent, and the protein was removed after centrifugation at 10,000 r/min for 15 min. A DEAE-Sepharose^TM^ Fast Flow column (2.5 cm × 30 cm) was used to receive 10 mL of a 5 mg/mL crude polysaccharide solution, which was prepared for SLMP purification. NaCl solutions of increasing concentrations (0, 0.1, 0.2, 0.4, and 0.6 mol/L) were used in sequential order for elution. An elution curve was generated, with the tube number on the x-axis and polysaccharide content (mg/mL) on the y-axis. The polysaccharide concentration was determined using the phenol sulfation method, and the spectrophotometric value at 490 nm was measured using a UV spectrophotometer. Using 200 mL of deionized water, the eluate fractions underwent further purification on a Sepharose^TM^ CL-6B column (2.6 cm × 60 cm). The fractions with the highest polysaccharide content were collected and stored by freeze drying.

### 2.6. Determination of Antioxidant Activity

Under the corresponding temperature conditions, the strength of antioxidant capacity was determined by the determination of the content of free radicals such as DPPH and ABTS in a certain concentration of polysaccharides. According to Wang Linqing’s method [20], the DPPH, ABTS, hydroxyl radical scavenging activity, and total reducing ability of SLMP3-1 were determined, but the polysaccharide concentration gradient (0.2–1.0 mg/mL) was changed to study the relationship between antioxidant activity and polysaccharide concentration. A curve was drawn with polysaccharide concentration as the horizontal coordinate and clearance rate as the vertical coordinate. Due to its clear strong antioxidant properties, Vc solution is often used as a positive control for antioxidant activity testing experiments.

### 2.7. Analysis of SLMPs

#### 2.7.1. Measurement of Molecular Weight

High-performance gel permeation chromatography (HPGPC) was conducted as previously described [21]. The columns G-5000 PWXL (7.8 × 300 mm, 10 μm) and G-3000 PWXL (7.8 × 300 mm, 5 μm) from Agilent Technologies were linked in series. About 1 mL of 0.02 M monopotassium phosphate solution was used to dissolve 2 g of SLMP3-1 powder. Monopotassium phosphate (0.02 M) was the mobile phase during elution. The same procedures were used to generate and evaluate dextran standards with known molecular weights.

#### 2.7.2. Measurement of Monosaccharide Composition

Monosaccharide content was ascertained by high-performance liquid chromatography (HPLC), as reported by Zhang et al. [22]. In summary, N_2_ was filled with an ampule holding 10 mg of the sample, and 2 mL of trifluoroacetic acid was then added. After the hydrolysate was dried at 70 °C in N_2_ and evaporation was performed thrice using methanol, the combination was allowed to interact at 70 °C for 2 h. Following the addition of 0.5 M 1-phenyl-3-methyl-5-pyrazolone methanol solution, 0.3 M HCl was used to neutralize the reaction mixture at room temperature.

#### 2.7.3. Fourier-Transform Infrared (FT-IR) Spectroscopy

As previously described [23], the FT-IR spectrum of the polysaccharide was obtained using an FT-IR spectrometer. After grinding, the polysaccharide was mixed with 140 mg of potassium bromide and pressed into tablets. The analysis was performed within 4000–400 cm^−1^, and the infrared spectra were recorded.

#### 2.7.4. ^1^H NMR Spectroscopy

As previously described [24], ^1^H NMR spectroscopy was performed. We dissolved 20 mg of SLMPs in 3.0 mL of deuterium oxide (99.8%). ^1^H NMR spectra were recorded using a Bruker Avance 400 MHz NMR spectrometer (Bruker Corporation, Billerica, MA, USA).

#### 2.7.5. Scanning Electron Microscopy Analysis

To further understand the effect of extraction methods on the structure of SLMPs, their microstructure was observed by scanning electron microscopy (SEM). As previously described [25], the microstructure of each material was characterized using an FEI Quanta 450 environmental SEM system (Thermo Fisher Scientific, Waltham, MA, USA) after the vacuum drying of the samples.

### 2.8. Statistical Analysis

Each experiment was conducted three times. Design-Expert software 13 was used to evaluate response-surface methodology (RSM), and a mathematical model was established based on the Box–Behnken central combination experimental design using Design-Expert 10.03 software.

## 3. Results and Discussion

### 3.1. Selecting the Optimal DES

**Figure 1:**
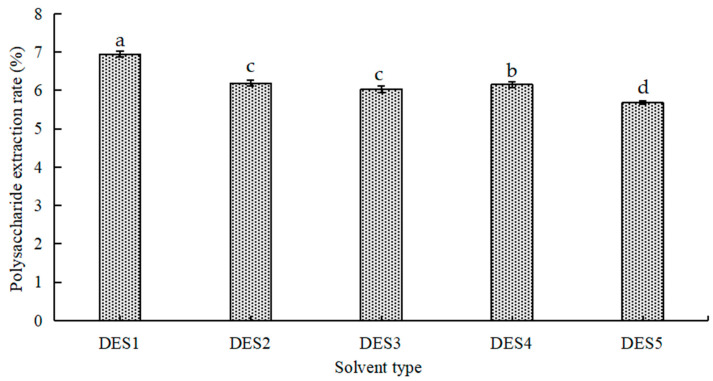
Effect of different DESs on polysaccharide extraction rate. (DES1) The mole ratio of choline chloride and ethylene glycol is 1:2. (DES2) The mole ratio of choline chloride and urea is 1:1. (DES3) The mole ratio of choline chloride and glycerol is 1:2. (DES4) The mole ratio of choline chloride and DL-malic acid is 1:1. (DES5) The mole ratio of choline chloride and citric acid is 1:1. Different letters represent different levels of significance, with ‘a’ indicating the highest significance and decreasing in order.

Different DESs with various chemical structures and physical properties can affect the activity of extracted polysaccharides [26]. Certain DESs, due to their polarity and viscosity, exert solubilizing effects that directly improve the extraction efficiency of target products. As shown in Figure 1, urea (DES2), two alcohols (DES1 and DES3), and two organic acids (DES4 and DES5) were selected as hydrogen-bond donors for the experiment. The results showed that the extraction rate of polysaccharides was lower when organic acid, urea, and glycerol were used as hydrogen-bond donors. Under the same conditions, the extraction yield of DES with ethylene glycol as a hydrogen-bond donor was the highest. This finding may be due to the combined diffusivity and polarity of ethylene glycol, which is similar to SLMPs, resulting in tighter hydrogen-bond interactions and increased polysaccharide extraction yield. Therefore, DES1 was chosen as the best extraction solvent, and the extraction process was improved in subsequent experiments.

### 3.2. Analysis of Single-Factor Experiment

#### 3.2.1. Effect of Ultrasonic Temperature on Polysaccharide Extraction Yield

**Figure 2:**
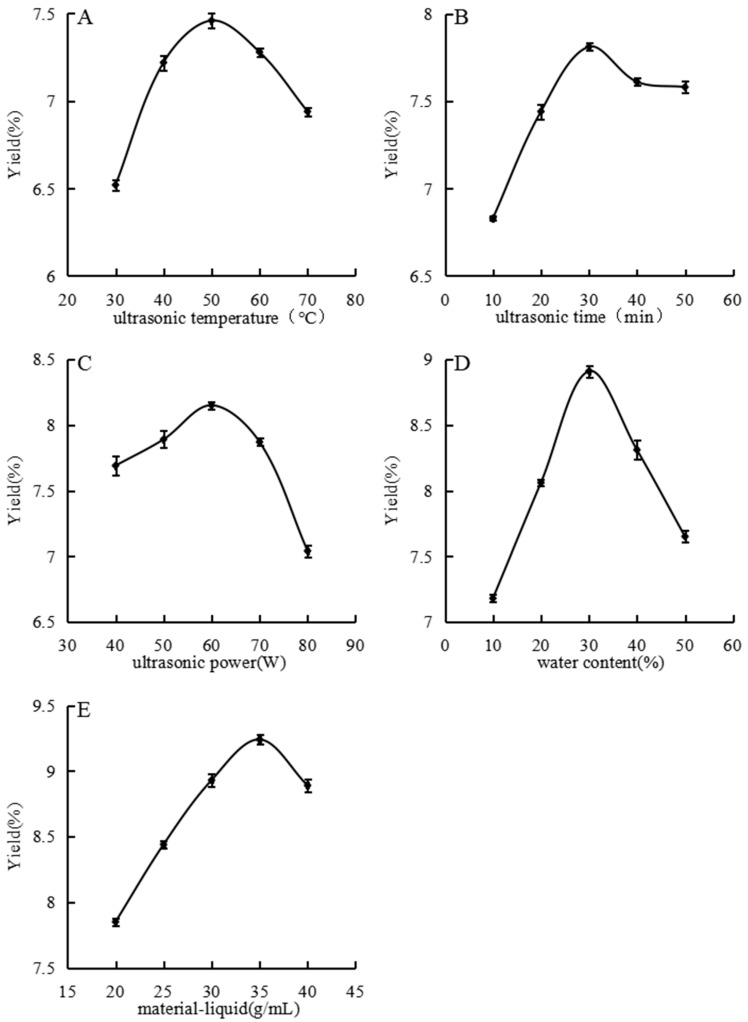
Effects of different ultrasonic temperature (**A**), ultrasonic time (**B**), ultrasonic power (**C**), water content (**D**), and material–liquid ratio (**E**) on extraction rate were studied.

Figure 2A illustrates the effect of ultrasonic temperature on polysaccharide extraction yield. The extraction yield increased and peaked at 50 °C with increased temperature from 30 °C to 50 °C. Beyond this range, the yield decreased with further increased temperature. At lower temperatures, insufficient interaction between solute and solvent led to a low extraction yield. Increasing the temperature accelerates molecular movement, thereby enhancing interactions, diffusion, and dissolution rates and significantly improving the extraction efficiency. However, temperatures above 50 °C can reduce the viscosity of the DES due to increased shear rates, potentially damaging its overall structure and thus reducing the polysaccharide extraction yield [27].

#### 3.2.2. Effect of Ultrasonic Time on the Polysaccharide Extraction Yield

As shown in Figure 2B, the extraction yield increased with time, peaked at 30 min, and then declined and plateaued. At 10 min, the extraction yield was low due to insufficient extraction time. Between 10 and 30 min, a significant amount of polysaccharides dissolved, rapidly increasing the yield. At 30 min, maximum extraction was likely achieved; extending the ultrasonic time further did not increase yield and may have degraded the polysaccharide structure or dissolved additional impurities. Consequently, the yield decreased [28].

#### 3.2.3. Effect of Ultrasonic Power on Polysaccharide Extraction Yield

Figure 2C shows the influence of ultrasonic power on extraction yield from 40 W to 80 W. The yield increased with power, peaking at 60 W, and then decreased with further increased power. Ultrasonic waves enhanced the extraction through cavitation and other effects, promoting vigorous molecular activity and collisions that released polysaccharides into the solvent. Beyond 60 W, the excessive mechanical shear from ultrasound may have damaged the polysaccharide chains, decreasing the polysaccharide content [29].

#### 3.2.4. Effect of Water Content on the Polysaccharide Extraction Yield

Figure 2D depicts the impact of water content on extraction yield. Adding water reduced the viscosity of the DES, facilitating molecular movement and reactions. The extraction yield increased with increased water content up to 30%, after which it declined. Below 30%, additional water improved polysaccharide dissolution and reduced solvent viscosity [30]. Exceeding the optimal water content may have hindered the interactions between the DES and solute, decreasing the yield.

#### 3.2.5. Effect of Material–Liquid Ratio on Polysaccharide Extraction Yield

As shown in Figure 2E, the extraction yield increased with increased material–liquid ratio, culminating at 1:35, as Figure 2E illustrates. However, with a further increased material–liquid ratio, the yield declined. Low ratios resulted in high viscosity and slow molecular movement, yielding low extraction efficiency. Increasing the solvent amount reduced the viscosity, enhanced the molecular interactions, and improved the extraction. Beyond the optimal ratio, additional solvent did not increase yield and led to resource wastage.

### 3.3. Response-Surface Experimental Design Analysis

RSM integrates experimental design with mathematical modeling for optimization. Based on single-factor experiments, central values were selected for the ultrasonic temperature (A), ultrasonic time (B), ultrasonic power (C), water content (D), and material–liquid ratio (E). As shown in Table 2 and Table 3, setting a five factor, three-level BBD resulted in 46 experimental runs, repeated three times to determine the optimal extraction conditions.

The following regression equation was obtained using software analysis:

Y = 9.36 − 0.1775A + 0.2325B − 0.3175C − 0.1944D + 0.1831E + 0.135A B − 0.225AC − 0.19AD + 0.52AE − 0.085BC + 0.3275BD + 0.0925BE − 0.43CE − 0.12DE − 0.5183A^2^ − 1.22B^2^ − 0.87C^2^ − 1.19D^2^ − 1.12E^2^

According to the regression equation results in Table 4, the model’s F-value of 17.76 and *p*-value of <0.0001 indicated significance. The five factors had linear and quadratic relationships with yield, showing close influence. The lack-of-fit *p*-value of 0.0807 (>0.05) suggested that the model accurately represented the data. The *R*^2^ value indicated a good fit, suitable for predicting the extraction process.

**Figure 3:**
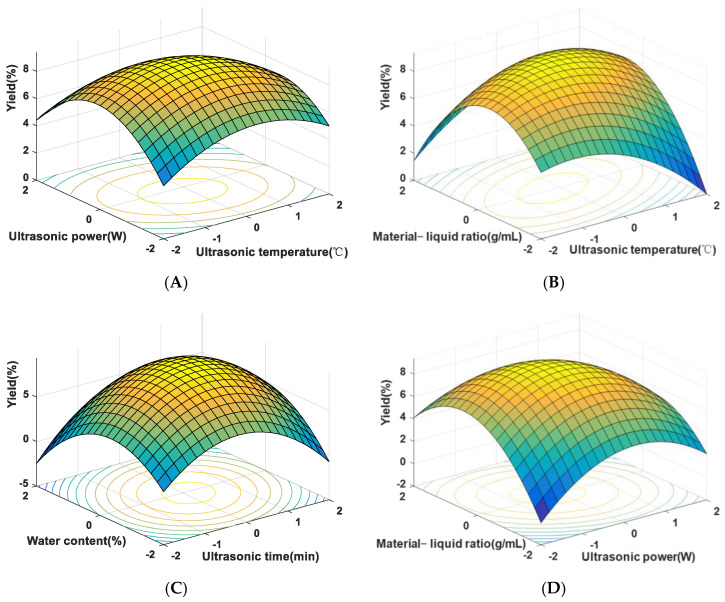
Response-surface plots for SLMP yield. (**A**) Effect of ultrasonic temperature and ultrasonic power on SLMP yield; (**B**) effect of material–liquid ratio and ultrasonic temperature on SLMP yield; (**C**) effect of water content and ultrasonic time on SLMP yield; (**D**) effect of material–liquid ratio and ultrasonic power on SLMP yield.

Response-surface plots helped visualize functional relationships, aiding in selecting optimal conditions. The steeper surfaces and rapid color changes in Figure 3A,C indicated significant effects on extraction yield. Elliptical contour lines in Figure 3B,D suggested significant interactions between factors. The optimal conditions determined were as follows: ultrasonic temperature 52 °C, ultrasonic time 31 min, ultrasonic power 57 W, water content 29%, and material–liquid ratio 1:36 g/mL, predicting a maximum yield of 9.39%. Verification experiments yielded an actual extraction yield of 9.25%, confirming the model’s feasibility.

### 3.4. Purification of SLMPs

**Figure 4:**
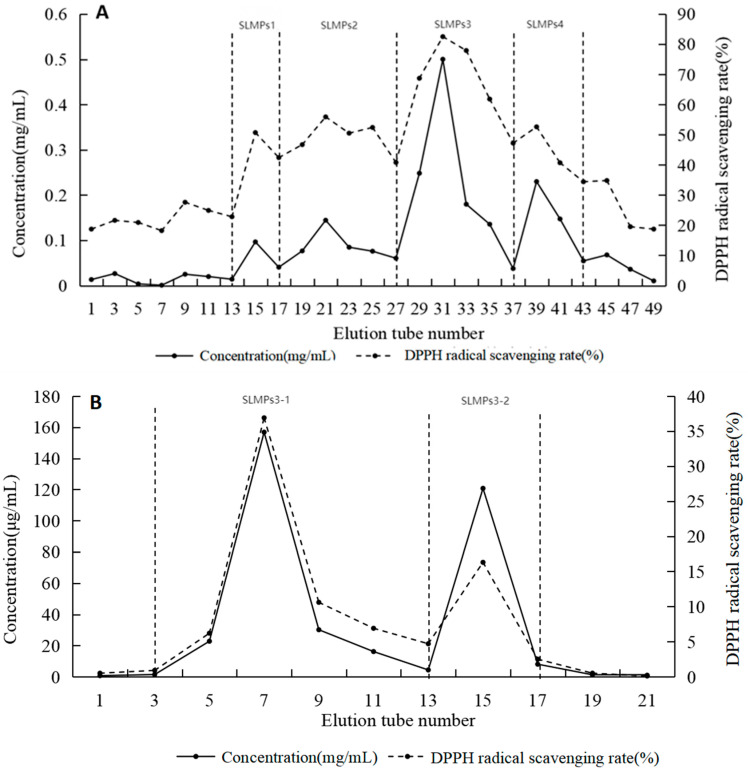
Purification of the polysaccharides. (**A**) DEAE-Sepharose^TM^ Fast Flow column gel filtration of SLMPs; (**B**) Sepharose^TM^ CL-6B gel filtration of SLMP3.

Polysaccharides with different charges were separated using DEAE-Sepharose™ Fast Flow ion-exchange chromatography. As shown in Figure 4A, four peaks were observed, with SLMP3 exhibiting the highest antioxidant activity in DPPH assays. SLMP3 was further purified using Sepharose™ CL-6B gel-filtration chromatography, resulting in a single peak named SLMP3-1 (Figure 4B). The purified samples were freeze-dried for preservation.

### 3.5. Analysis of Antioxidant Activity of Polysaccharides

The human body produces free radicals, and excessive free radicals affect people’s health. Natural plant polysaccharides can mitigate these effects by chelating metal ions, scavenging free radicals, and donating electrons [31].

#### 3.5.1. DPPH Radical-Scavenging Activity

**Figure 5:**
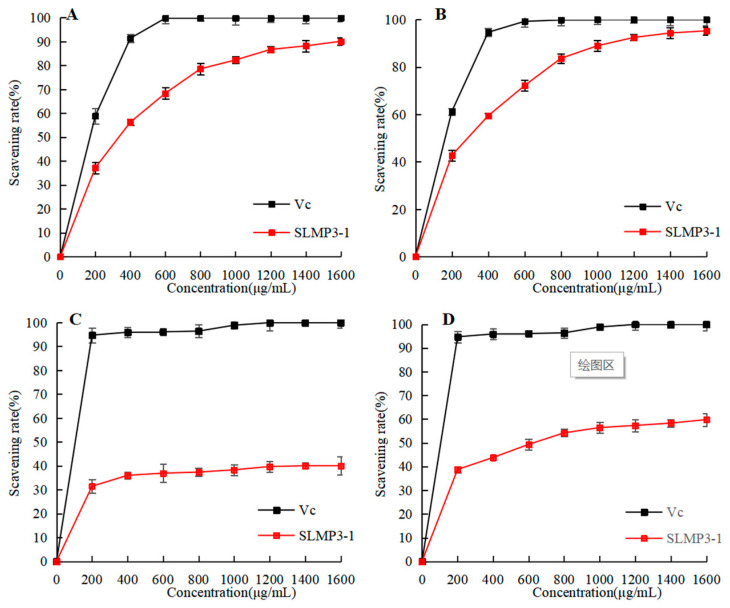
Antioxidant activity of SLMP3-1. (**A**) DPPH radical-scavenging activity; (**B**) ABTS radical-scavenging activity; (**C**) hydroxyl radical-scavenging activity; (**D**) total reducing antioxidant power.

The rate at which DPPH radicals can be scavenged is a widely used technique in evaluating the antioxidant capacity of various substances. Figure 5A illustrates a noteworthy dose–response connection between the DPPH radical-scavenging rate and the concentration of SLMP3-1. Considering the strong antioxidant activity of Vc solution, Vc solution with the same concentration gradient was selected as the positive control group. The antioxidant activity of Vc solution increases with the increase in concentration, and the trend of changes in the antioxidant activity of polysaccharides is the same as that of Vc solution. The IC_50_ value was 327 μg/mL. According to Norouzi Akbar [32], the IC_50_ value of SLMP3-1 is similar to that of the polysaccharide from *Sargassum angustifolium* (PSA, IC_50_ = 320 μg/mL), whereas this study illustrated that PSA had strong antioxidant activity, so SLMP3-1 also had strong antioxidant activity.

#### 3.5.2. ABTS Radical-Scavenging Activity

ABTS free-radical-scavenging rate is another common measure of antioxidant activity. As shown in Figure 5B, the trend for ABTS cationic radical-scavenging capacity was similar to that for DPPH radical-scavenging capacity. SLMP3-1 also had a concentration-dependent relationship for ABTS cationic radical-scavenging capacity, with a semi-inhibitory concentration of 291 μg/mL. According to Bhuyar Prakash [33], the IC_50_ of polysaccharides from *Padina Gymnospora* was 2.565 mg/mL, which was significantly higher than that of SLMP3-1. This finding was in line with the DPPH radical-scavenging experiment results.

#### 3.5.3. Hydroxyl Radical-Scavenging Activity

The hydroxyl radical is often used as an index to simulate oxidative stress in vivo. As shown in Figure 5C, the hydroxyl radical-scavenging rate was related to the polysaccharide concentration. This may be due to the strong attraction of intermolecular and intramolecular hydrogen bonds, which can inhibit the hydroxyl reaction and reduce the ability to scavenge hydroxyl radicals [34].

#### 3.5.4. Total Reducing Antioxidant Power

Reducibility usually results in the destruction of the free-radical chain by the transfer of a hydrogen atom. By measuring the absorbance value, the findings may be ascertained; i.e., a greater antioxidant activity corresponds with a higher absorbance value. According to Figure 5D, the pattern of the curve was consistent with the trend of hydroxyl radical-scavenging capacity. The reduction ability of SLMP3-1 increased with increased polysaccharide concentration [35].

### 3.6. Composition and Structure Analysis

#### 3.6.1. Molecular Weight

**Figure 6:**
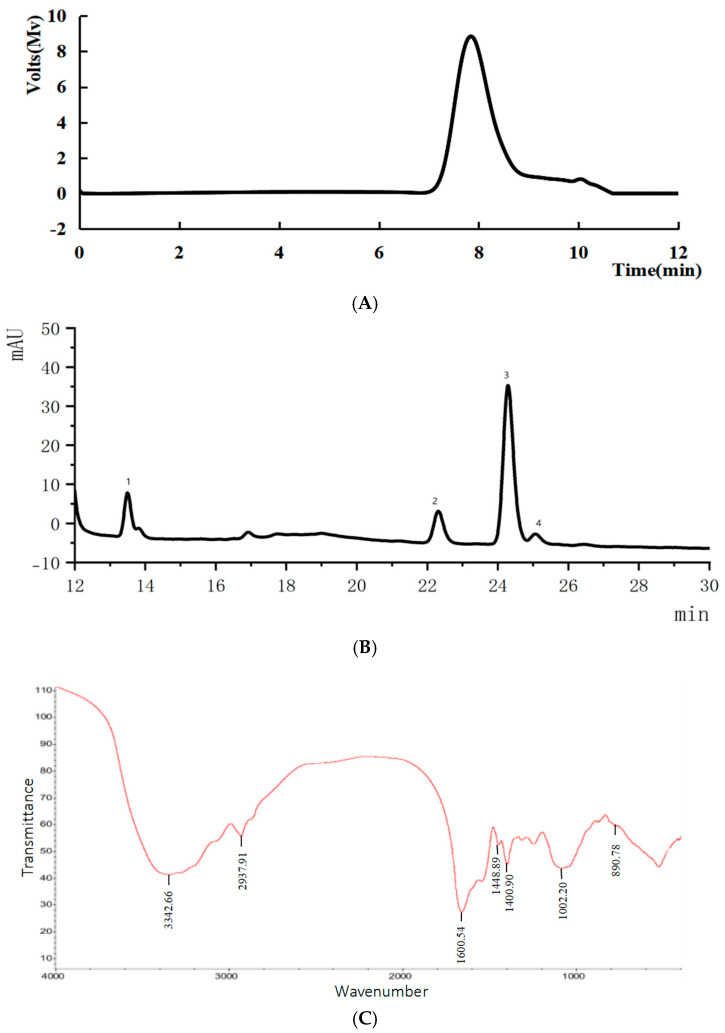

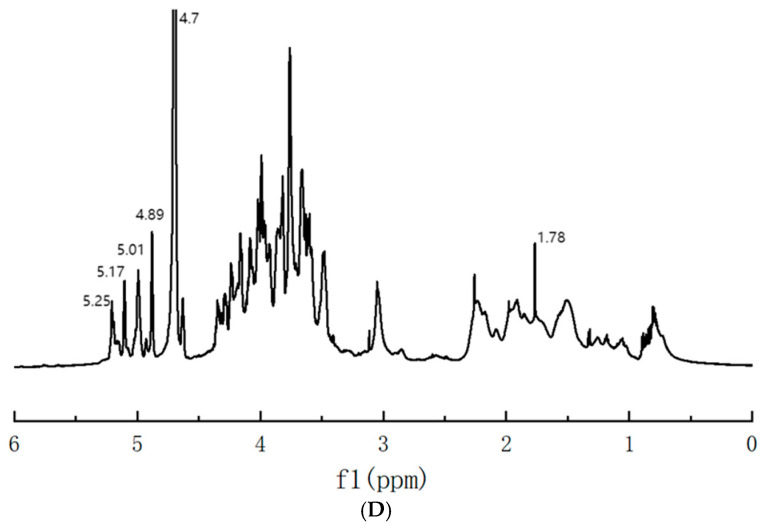
(**A**) Molecular weight of SLMP3-1; (**B**) monosaccharide composition of SLMP3-1; (**C**) FT-IR spectra of SLMP3-1; (**D**) NMR spectral analysis of SLMP3-1.

Figure 6A shows a symmetrical peak at approximately 8 min, indicating the polysaccharide’s molecular weight of 20.7 kDa.

Low-molecular-weight polysaccharides demonstrated enhanced antioxidant activity. This potent activity can be attributed to the presence of reductive groups, specifically amino and hydroxyl groups. They are known to interact favorably with oxidants and reactive radicals [36], classifying SLMP3-1 as a potent antioxidant.

#### 3.6.2. Monosaccharide Composition

HPLC analysis (Figure 6B) revealed that SLMP3-1 comprised glucose, mannose, arabinose, and galactose in molar ratios of 6.36:3.13:28.63:1, respectively. Polysaccharides rich in Glc, Gal, and Ara were correlated with strong antioxidant activity [37], consistent with the observed properties of SLMP3-1.

#### 3.6.3. Infrared (FT-IR) Spectroscopy Analysis

The FT-IR spectrum of SLMP3-1 (Figure 6C) had prominent absorption peaks of polysaccharides within the 400–4000 cm⁻^1^ range. O–H stretching, C–H stretching of methyl and methylene groups, and C=O stretching in acylamino groups were shown by the peaks at 3342.66, 2937.91, and 1660.54 cm⁻^1^ [38,39]. The peaks located at 1448.89 and 1400.90 cm⁻^1^ signified symmetric stretching of the -COO- groups and C–O vibrations. The peak at around 1000 cm⁻^1^ corresponded with C–O stretching in pyran rings [19]. These features are typical of polysaccharides.

#### 3.6.4. ^1^H NMR Spectroscopy Analysis

In the ^1^H NMR spectrum (Figure 6D), anomeric protons in α- and β-configured pyranose rings appeared at δ 5.1–5.8 and δ 4.3–4.8 ppm, respectively [40]. SLMP3-1 showed signals between δ 4.80 and 5.25 ppm, indicating α-configuration. Peaks at δ 4.89, 5.01, 5.14, and 5.25 ppm corresponded with α-Gal, α-Glc, α-Man, and α-Ara, respectively [41]. In glycosidic linkages, signals ranging from δ 3.2 to 4.5 ppm indicated overlapping H-2 to H-6 protons [42], in line with the FT-IR and monosaccharide analysis results.

#### 3.6.5. SEM Analysis

**Figure 7:**
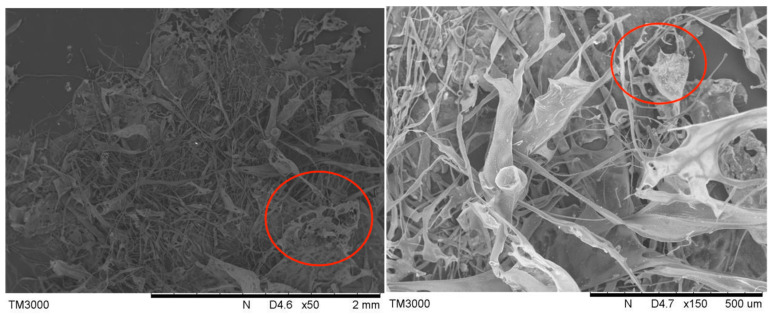
SEM images of SLMPs extracted by UAE.

The polysaccharide samples were dissolved in a liquid phase, such as diluted water, and their distribution and structure were analyzed by SEM. As shown in Figure 7, the microstructure of SLMPs extracted by the DES-UAE method significantly changed, with more visible wrinkles and pores on the surface of the SLMPs due to the cavitation effect of the ultrasound, showing an overall transparent state. A scanning electron microscope can detect the spherical, flaky, and porous structures of antioxidant polysaccharides [43]. The porous structure plays a role in chelating ferrous ions, enhancing the antioxidant activity of the polysaccharides [44]. Therefore, after the cell wall was destroyed, the exudation of polysaccharides was enhanced, and the low eutectic solvent showed stronger permeability. These findings indicated the DES-UAE method was effective for extracting SLMPs and can have wide-ranging applications.

## 4. Conclusions

This study developed a new deep eutectic solvent–ultrasound-assisted extraction (DES-UAE) process based on choline chloride/ethylene glycol (1:2). The optimal process parameters were obtained through response-surface methodology optimization: ultrasonic temperature of 52 °C, time of 31 min, power of 57 W, water content of 29%, solid–liquid ratio of 1:36 g/mL, and polysaccharide yield of 9.25% (*w*/*w*). Structural characterization indicates that the obtained SLMP3-1 has a low molecular weight (20.7 kDa) and a porous microstructure, and its antioxidant activity is closely related to its structural characteristics.

This technology combines high efficiency and environmental friendliness, providing a theoretical basis for the industrial production of algal residue polysaccharides. Meanwhile, this research on raw materials has universality and can be extended to other plant polysaccharides, providing new ideas for the research, extraction, and exploration of polysaccharides. The extracted antioxidant polysaccharides can provide new strategies for functional foods and disease prevention and treatment and have important health industry value.

## Figures and Tables

**Table 1:** Preparation of DES species.

Number	Makeup	Molar Ratio
DES1	Choline chloride/ethylene glycol	1:2
DES2	Choline chloride/urea	1:1
DES3	Choline chloride/glycerol	1:2
DES4	Choline chloride/DL-malic acid	1:1
DES5	Choline chloride/citric acid	1:1

**Table 2:** Response-surface test factor levels and coding.

Encodings	A (Ultrasonic Temperature, °C)	B (Ultrasonic Time, min)	C (Ultrasonic Power, W)	D (Water Content, %)	E (Material–Liquid Ratio, g/mL)
−1	40	20	50	20	30
0	50	30	60	30	35
1	60	40	70	40	40

**Table 3:** Experimental design and results.

Number	A (Ultrasonic Temperature, °C)	B (Ultrasonic Time, min)	C (Ultrasonic Power, W)	D (Water Content, %)	E (Material–Liquid Ratio, g/mL)	Yield (%)
1	−1	−1	0	0	0	7.59
2	1	−1	0	0	0	7.25
3	−1	1	0	0	0	7.85
4	1	1	0	0	0	8.05
5	0	0	−1	−1	0	7.81
6	0	0	1	−1	0	6.94
7	0	0	−1	1	0	7.89
8	0	0	1	1	0	7.56
9	0	−1	0	0	−1	6.51
10	0	1	0	0	−1	6.93
11	0	−1	0	0	1	6.62
12	0	1	0	0	1	7.41
13	−1	0	−1	0	0	7.74
14	1	0	−1	0	0	7.91
15	−1	0	1	0	0	8.09
16	1	0	1	0	0	7.36
17	0	0	0	−1	−1	7.23
18	0	0	0	1	−1	8.31
19	0	0	0	−1	1	7.57
20	0	0	0	1	1	6.56
21	0	−1	−1	0	0	7.44
22	0	1	−1	0	0	7.64
23	0	−1	1	0	0	6.97
24	0	1	1	0	0	7.13
25	−1	0	0	−1	0	7.89
26	1	0	0	−1	0	7.97
27	−1	0	0	1	0	7.55
28	1	0	0	1	0	6.87
29	0	0	−1	0	−1	7.02
30	0	0	1	0	−1	6.95
31	0	0	−1	0	1	8.61
32	0	0	1	0	1	6.82
33	−1	0	0	0	−1	8.67
34	1	0	0	0	−1	6.86
35	−1	0	0	0	1	7.97
36	1	0	0	0	1	8.24
37	0	−1	0	−1	0	7.15
38	0	1	0	−1	0	6.89
39	0	−1	0	1	0	6.35
40	0	1	0	1	0	7.40
41	0	0	0	0	0	9.48
42	0	0	0	0	0	9.21
43	0	0	0	0	0	9.49
44	0	0	0	0	0	9.23
45	0	0	0	0	0	9.56
46	0	0	0	0	0	9.18

**Table 4:** Results of regression equations.

Source	Sum of Squares	Degrees of Freedom	Variance	F-Value	*p*-Value
Model	31.20	20	1.56	17.76	<0.0001
A	0.5041	1	0.5041	5.74	0.0244
B	0.8649	1	0.8649	9.85	0.0043
C	1.61	1	1.61	18.37	0.0002
D	0.6045	1	0.6045	6.88	0.0146
E	0.5366	1	0.5366	6.11	0.0206
AB	0.0729	1	0.0729	0.8301	0.3710
AC	0.2025	1	0.2025	2.31	0.1414
AD	0.1444	1	0.1444	1.64	0.2115
AE	1.08	1	1.08	12.32	0.0017
BC	0.0289	1	0.0289	0.3291	0.5713
BD	0.4290	1	0.4290	4.89	0.0365
BE	0.0342	1	0.0342	0.3897	0.5381
CD	0.0000	1	0.0000	0.0000	1.0000
CE	0.7396	1	0.7396	8.42	0.0076
DE	0.0576	1	0.0576	0.6559	0.4257
A^2^	2.34	1	2.34	26.70	<0.0001
B^2^	12.92	1	12.92	147.10	<0.0001
C^2^	6.61	1	6.61	75.22	<0.0001
D^2^	12.38	1	12.38	140.92	<0.0001
E^2^	10.90	1	10.90	124.10	<0.0001
Residual	2.20	25	0.0878		
Lack of Fit	2.05	20	0.1026	3.59	0.0807
Pure Error	0.1431	5	0.0286		
Total	33.40	45			
Std. Dev.	0.2964				
Mean	7.65				
C.V.% R^2^ Adj-R^2^	3.87 0.9343 0.8817				

## Data Availability

The original contributions presented in this study are included in the article. Further inquiries can be directed to the corresponding author.
